# Poliovirus Immunity among Children Aged 6–11 and 36–48 Months in 14 Polio High-Risk Provinces of Afghanistan: A Health-Facility-Based Study

**DOI:** 10.3390/vaccines10101726

**Published:** 2022-10-16

**Authors:** Sajid Bashir Soofi, Maureen Martinez, Noha H. Farag, William S. Hendley, Derek Ehrhardt, Imran Ahmed, Imtiaz Hussain, William Weldon, Ahmed M. Kassem

**Affiliations:** 1 Centre of Excellence in Women and Child Health, Aga Khan University, Karachi 74800, Pakistan; 2 The Centers for Disease Control and Prevention, Atlanta, GA 30329-4027, USA

**Keywords:** polio, children, serosurvey, health facilities, Afghanistan

## Abstract

Afghanistan is one of two countries where wild poliovirus (WPV) type 1 remains endemic. We conducted a facility-based cross-sectional survey of antipoliovirus antibodies in children in 14 provinces of Afghanistan. The provinces were selected based on programmatic priorities for polio eradication. Children aged 6–11 and 36–48 months attending outpatient clinics were enrolled in the study. We collected venous blood, isolated serum, and conducted neutralization assays to detect poliovirus neutralizing antibodies. A total of 2086 children from the 14 provinces were enrolled. Among the enrolled children, 44.3% were girls; the median age in the 6–11-month group was 9.4 months, and in the 36–48-month group, it was 41.8 months. The most common spoken language was Pashtu (70.8%). Eighty-two percent of children were fully immunized against all the diseases in the vaccination schedule of Afghanistan. In the children aged 6–11 months, seroprevalence to poliovirus type 1 (PV1) was 96.5% and seroprevalence to poliovirus type 3 (PV3) was 93%; in children aged 36–48 months, seroprevalence to PV1 was 99.5% and to PV3 was 98%. Antipoliovirus antibody prevalence for poliovirus type 2 (PV2) was 70.5% in the younger group compared with 90.9% in the older children. Children from Herat and Laghman provinces had almost 100% seroprevalence to PV1, and other provinces also had high prevalence, ranging from 92.0% to 99.0%. A similar finding was seen for antibodies against PV3, ranging from 88% to 100% by province. On the contrary, antibodies to PV2 were low, ranging from 53% for children in the Khost province to around 89% in Kunduz. There was a cluster of 18 seronegative children in the Nuristan province. Overall, the polio eradication program of Afghanistan has been successful in achieving high seroprevalence of poliovirus neutralizing antibodies in the parts of the country included in this study.

## 1. Introduction

Since the launch of the Global Polio Eradication Initiative in 1988, the number of cases of paralytic poliomyelitis plummeted by more than 99 percent worldwide by 2000 [1,2,3]. In 1988, wild poliovirus (WPV) types 1, 2, and 3 were circulating in 125 countries [4,5]. In 2018, only WPV type 1 circulation persisted and was confined to the two endemic countries, Afghanistan and Pakistan (The last WPV1 case in Nigeria was detected in 2016; interruption of endemic WPV transmission in the World Health Organization African Region was certified in August 2020. In 2022, one paralytic WPV1 case was confirmed in Malawi, and one paralytic WPV1 was detected in Mozambique outside the two endemic countries of Afghanistan and Pakistan). The WPV type 1 poliovirus detected in Malawi was genetically linked to a Pakistan sequence detected in 2020 in the province of Sindh [6]. Moreover, the circulating vaccine-derived poliovirus type 2 (cVDPV2) was also detected recently in the U.K. in May 2022 and in New York in July 2022 [7]. The current polio epidemiology in the two endemic countries poses an inimitable challenge for global eradication as these countries are being affected by ongoing endemic WPV1 and cVDPV2 transmission. Between January 2020 to November 2022, Afghanistan reported 60 cases of WPV1 and 351 cases of cVDPV2 [8].

In Afghanistan, armed conflict and insecurity, coupled with frequent population movements, have impeded access by children to vaccinations [9]. Despite this, national routine vaccination coverage rates have increased from 35% in 2001 to 66% in 2011 [10], as estimated by the WHO/UNICEF. The most recent available survey results from the Afghanistan Health Survey 2018 (AHS) estimate the national immunization coverage at 50% among children aged 12–23 months [11] in the country. The routine immunization schedule in Afghanistan includes oral poliovirus vaccine (OPV) given at birth; 6, 10, and 14 weeks of age; and inactivated poliovirus vaccine (IPV), introduced in 2016 into the routine immunization schedule, administered at 14 weeks of age [12], as presented in Table 1 below.

The estimated routine immunization coverage proportion for the third OPV dose by age 11 months was 60% in 2015–2017; for IPV, it was 65% in 2017 [13]. In April 2016, a globally synchronized switch from trivalent OPV (tOPV; types 1, 2, and 3 Sabin strains) to bivalent OPV (bOPV; types 1 and 3 Sabin strains) [14] resulted in the cessation of use of Sabin OPV type 2 strain in Afghanistan in routine immunization.

Afghanistan’s polio National Eradication Action Plan serves as a guide for all polio eradication activities, and since its first adoption during 2012–2013, there has been a significant reduction in the geographic spread of the virus through the consistent and well-tracked implementation of immunization campaigns [15]. In 2016, Emergency Operation Centers (EOCs) were established at the national and regional levels to strengthen and coordinate the efforts of all partners involved. Four National Immunization Days (NIDs) targeting 10 million children and six subnational NIDs targeting six million children were conducted in 2017, along with three mop-up campaigns [16]. In 2018, a total of three NIDs and six subnational NIDs were conducted. According to the World Health Organization’s independent postcampaign monitoring data from the Afghanistan office, coverage in each campaign during 2017 was more than 94%. The number of inaccessible children declined from 391,000 in 2017 to 138,000 in March 2018 but drastically increased in May 2018 when 996,326 children were reported inaccessible due to vaccination bans, mostly in the southern region. A special vaccination strategy was adopted in 2017 to reach seasonal nomadic populations in the southeastern region. Children on the move, especially those returning from Pakistan and Iran, were immunized by crossborder teams (CBTs) and permanent transit teams (PTTs) using OPV and IPV [17]. Five provinces in Afghanistan were identified as high-risk for sustaining poliovirus transmission based on poliovirus epidemiology, access to supplementary immunization activities (SIAs), estimated population immunity, and the presence of refugees and internally displaced persons (IDPs). These provinces are Kandahar, Helmand, Nangarhar, Kunar, and Farah [18]. In 2017, when this study was being planned, there were 14 cases of WPV1 reported, and 21 cases were reported in 2018 across Afghanistan, with most cases in 2017 and 2018 in the high-risk provinces in the eastern and southern regions of the country. Moreover, the WPV1 cases in the country are constantly rising. Between January 2020 to November 2021, 60 WPV1 cases were reported in Afghanistan, with most cases clustered in the eastern and southern regions of the country [8].

Serological surveys of antipoliovirus neutralizing antibodies have been carried out in many countries as a tool to evaluate program performance, assess population immunity gaps, and identify areas of high risk for poliovirus transmission [19,20,21,22]. The poliovirus immunity profiles of young children in China, Egypt, Indonesia, Madagascar, and India [23] have provided country-specific results that have led to programmatic actions and an immunity benchmark for the eradication initiative. Similar studies were previously conducted in Afghanistan to assess the seroprevalence among children under five years of age. In 2013, a study assessed poliovirus immunity among children under five years in the accessible areas of Afghanistan [24]; in 2017, a facility-based study focusing on children aged 6–11 and 36–48 months in polio high-risk areas in Kandahar Province [25] was carried out. These studies helped to analyze the trend of seroprevalence among the target population in the country. Providing scientific evidence about the existing level of seroprevalence, vaccination coverage, and its effectiveness in Afghanistan will help in designing informed decisions to address the existing challenges in poliovirus eradication efforts, enabling national and international stakeholders to devise measures to further improve the quality and effectiveness of both routine and supplementary immunizations activities and to help evaluate the risk of future outbreaks of WPV1 and cVDPV2 in the country.

Considering the importance of quantifying the levels of serological protection against poliovirus, we conducted an antipoliovirus neutralizing antibody serological survey among children who were visiting selected health facilities in 14 provinces of Afghanistan. The provinces selected were high risk for polio or in proximity to those areas with potential immunity gap concerns.

## 2. Materials and Methods

### 2.1. Study Design and Study Population

**Figure 1:**
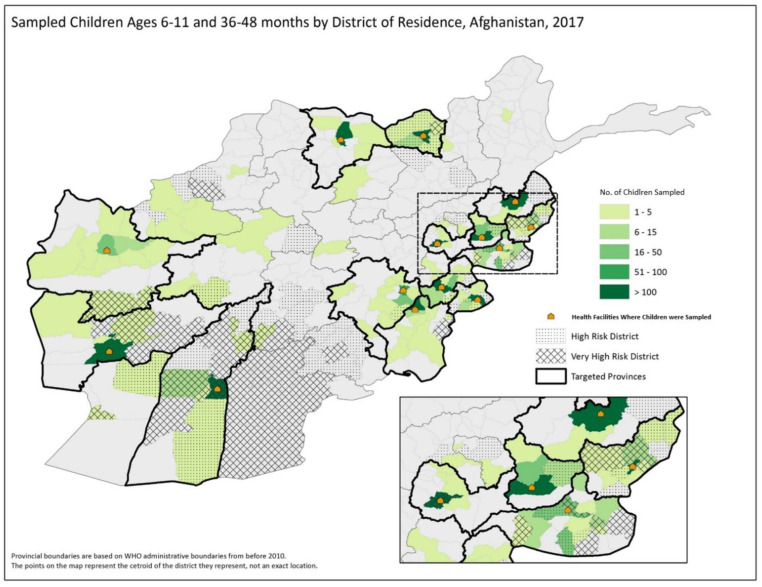
Provinces included in the serosurvey, Afghanistan, 2017. The map call-out shows details of the eastern region of Afghanistan for ease of viewing.

A facility-based cross-sectional survey was conducted in 14 polio program priority provinces of Afghanistan. The provinces were selected based on programmatic priority for polio, chronic accessibility challenges, poor campaign quality, presence of special populations (refugees, returnees, and nomads), proximity to the border with Pakistan, and concerns about program quality (Figure 1). A central health facility in the province was selected as the study site (Table 2). The choice of regional and provincial hospitals in the selected provinces was based on reported referral patterns to provide a reasonable representation of the catchment population.

Children aged 6–11 and 36–48 months attending the outpatient clinics with an adult caretaker at the selected health facilities were approached for participation in the study. A purposive sampling technique was used for the selection of children from both age groups at each health facility. Children attending a selected health facility for ailments unrelated to polio and accompanied by a primary adult caregiver were eligible for enrollment. Before enrollment in the study, a detailed history was taken, and a physical examination was performed on each child to ascertain eligibility. Exclusion criteria encompassed severe acute or chronic illness requiring immediate medical attention, including immunodeficiency and neoplasms.

Informed consent was obtained from caretakers of all eligible children enrolled in this study. In case of refusal to participate, parents and caregivers were asked to answer a few questions (e.g., age of child; district and village of residence; population group, e.g., nomad, returnee, refugee, etc.; travel history; and OPV and IPV immunization status of the child (routine immunization (RI) and SIAs). These data were collected to assist in quantifying the possible effects of the nonresponse rate on the serosurvey results. Enrolment and sample collection were performed during 25 October–20 November 2017. For provincial level estimates, the required sample size in each age group was calculated assuming seroprevalence of 90%, precision of ±6%, and alpha = 0.05. A sample size of 140 children in each province (70 in each age group) was calculated and enrolled in the survey after obtaining the consent of a parent or primary caregiver.

### 2.2. Training of the Field Team

Training of locally hired field teams was conducted over three days in Kabul. The field team was specially trained to collect data using the structured questionnaire on handheld devices, and phlebotomists were trained in phlebotomy procedures, and sample labelling, handling, and transportation. For conceptual clarity of the survey, team field pilot testing was conducted in the Indira Gandhi Hospital in Kabul. All steps of the survey were followed in the pilot exercise, including the data collection, phlebotomy, sample handling and transportation, and cold chain guidelines.

### 2.3. Data Collection

A structured questionnaire was developed to collect data on demography, socioeconomic status (SES), comorbidities, nutritional status, and vaccination history. Vaccination history for OPV and IPV received through routine immunization was assessed from vaccination cards when available or by parental/caregiver recall if cards were not available. The number of OPV or IPV doses received through campaigns was always obtained by parental/caregiver recall, as no documentation exists.

Data at the facility were electronically collected using handheld devices running Android Operating System. Android Version 5.1.1 created by Google, 1600 Amphitheatre Parkway Mountain View, CA 94043, USA was used. A customized data collection application was developed in-house using Java language with MySQL, and SQLite was running at the backend for data storage. The application offered built-in features for real-time data quality checking and identification of missing information, inconsistencies, outliers, and other possible errors and omissions. The users were prompted for any such errors at the time of data collection to ensure quality data.

### 2.4. Laboratory Methodology

Trained phlebotomists collected 2 mL of peripheral blood from each subject using standard venipuncture techniques. After clotting and centrifugation, sera were separated, transferred into labeled sterile cryovials, immediately stored in a cold box with ice packs, and transported to the nearest provincial or regional hospital for temporary storage in the expanded program on immunization (EPI) cold chain. After completion of field activities, samples were transferred under reverse cold chain conditions to Kabul for further transportation to the Aga Khan University (AKU) for further transportation to the AKU Nutrition Research Laboratory in Karachi. In the laboratory, two aliquots were prepared, one for backup and one for transport to the Centers for Disease Control and Prevention in Atlanta, USA, for poliovirus neutralization assay testing [15]. Seroprevalence, defined as the proportion of subjects with titers ≥3 [1/dil], was calculated for each poliovirus serotype (PV1, PV2, and PV3).

### 2.5. Statistical Analysis

Frequencies and percentages were calculated for categorical variables to describe the characteristics of the participants. Seroprevalence estimates by PV type were calculated as proportions with their 95% confidence intervals. All analyses were completed using Stata Version 12 [26], sourced from StataCorp LLC, College Station, TX, USA.

### 2.6. Ethical Review

This study received approval from the Ethical Review Committees of the World Health Organization, Aga Khan University, and the National Bioethics Committee of the Government of Pakistan and from the Institutional Review Board of the Ministry of Public Health, Afghanistan. The Center for Global Health, Centers for Disease Control and Prevention Office of Human Subjects Research Protection, deemed this study as nonresearch and deferred approval to the Institutional Review Board of Aga Khan University (CDC project ID 0900f3eb819a146a).

## 3. Results

Blood samples were obtained from 2086 (99%) eligible children from both age cohorts out of 2104 children who were approached to participate in the study. There were 44.3% girls among the enrolled children (44% in the age group of 6–11 months and 44.5% in the age group of 36–48 months); the median age in the 6–11 months group was 9.4 months and that in the 36–48 months group was 41.8 months. Table 3 shows the demographic indicators, sex, and vaccination history of the study population. The respondents could report more than one spoken language; the most common was Pashtu (70.8%), followed by Dari (30.5%). Around 82% of children were fully immunized against all the diseases in the vaccination schedule of Afghanistan: 92% in the older age group and 72% younger age group.

**Figure 2:**
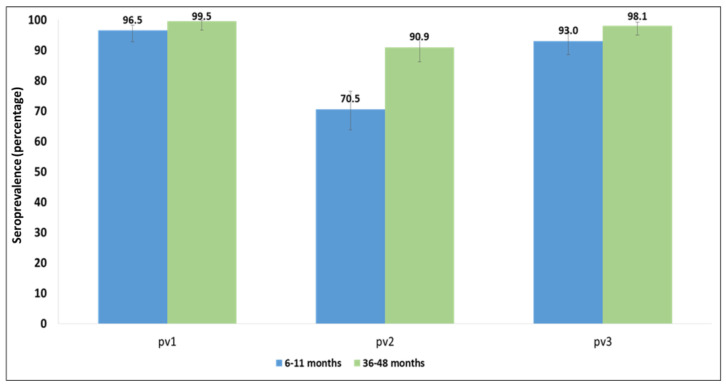
Seroprevalence by age group and poliovirus serotype, Afghanistan, 2017. Percentage is shown above each condition.

In children aged 6–11 months, seroprevalence to poliovirus type 1 (PV1) was 96.5%, and seroprevalence to poliovirus type 3 (PV3) was 93.0%; in children aged 36–48 months, seroprevalence to PV1 was 99.5% and that to PV3 was 98.1% (Figure 2). Antipoliovirus neutralizing antibody prevalence levels for poliovirus type 2 (PV2) were much lower among the study population: 70.5% in younger compared with 90.9% in older children.

**Figure 3:**
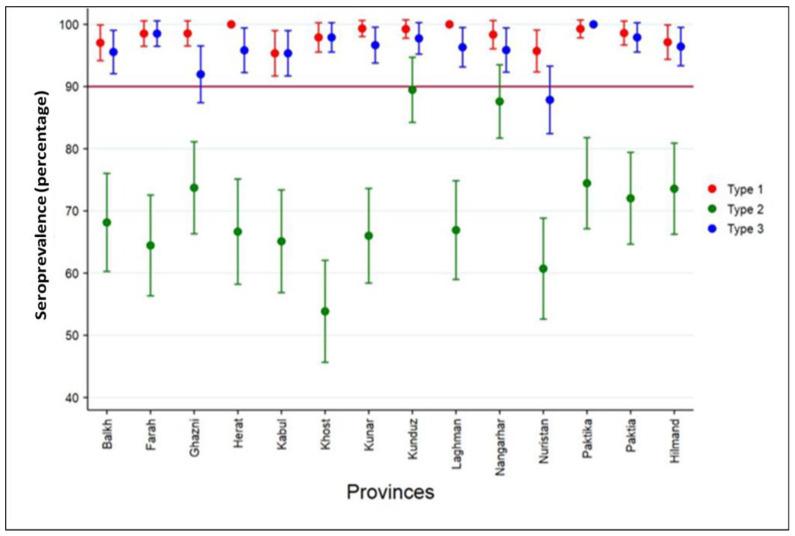
Seroprevalence by poliovirus serotype and province of residence, Afghanistan, 2017.

Children from Herat and Laghman provinces had almost 100% seroprevalence to PV1, whereas children from other provinces also had high prevalence, 92–99% (Figure 3). High seroprevalence was similarly observed against PV3, 88–100%. Neutralizing antibody prevalence levels to PV2 were lower, ranging from 53% for Khost province to 89.9% for Kunduz. There was a cluster of 18 children who were seronegative to PV1 or PV3 in Poruns district, Nuristan province.

## 4. Discussion

This survey provides critical information on the immunity status of children against poliovirus in high-risk provinces of Afghanistan. The seroprevalence of antipoliovirus neutralizing antibodies was >95% for both age groups for PV1 and for PV3 in the older age group. The PV3 antipoliovirus neutralizing antibodies level among the younger age group was 93%. Neutralizing antibody prevalence to PV2 was ~20% lower among children aged 6–11 months compared with those aged 36–48 months.

The lower PV2 seroprevalence among the younger study population is explained by the inclusion of children in the younger age group who were born after the switch from tOPV to bOPV. Children born before the switch should not have received any type 2 OPV, but they had all received one IPV dose at 14 weeks of age or after. Former studies on immunogenicity of one dose of IPV administered at 14 weeks and bOPV at 6, 10, and 14 weeks showed a type 2 seroconversion rate of 69–80% [27]. In this study, older Afghan children born after the switch had a PV2 seroconversion rate of 70.5%. Circulating vaccine-derived polioviruses (cVDPVs) can emerge in settings with low population immunity and cause paralysis. Type-2-naïve children who were born after the switch from tOPV to bOPV and who lived in areas with chronically low routine and supplementary polio immunization coverage are at particularly high risk of cVDPV2 infection.

The findings of this study are consistent with the results of a seroprevalence survey conducted in Kandhar province in 2017 [25], which recorded a high seroprevalence for PV1 and PV3 among children of both age groups (6–11 months and 36–48 months) and a low seroprevalence for PV2 among the younger age cohort compared with the older children. A similar study in polio high-risk areas of Pakistan in 2016 recorded high seroprevalence for PV1 and PV3 and low for PV2 among younger children [28]. Another study conducted in Pakistan in 2016 to quantify immunity and field efficacy of type-2-containing polio vaccines after cessation of the trivalent oral polio vaccine revealed that type 2 seroprevalence was 50% among children 6–11 months old born after the withdrawal of tOPV within April 2016 [29]. Similar findings were recorded in a study that assessed population immunity in a persistently high-risk area for wild poliovirus transmission in Western Uttar Pradesh of India in 2007 [30]. Nevertheless, pockets of underimmunized children remain in high-risk areas, which may continue to transmit WPV within the two neighboring countries. In this survey, we identified a cluster of seronegative children in the district Poruns of Nuristan province. Similar clusters may exist elsewhere. In a previous study conducted in 2013 to assess the polio virus immunity among children under five years of age in Afghanistan, a cluster of 14 seronegative children was reported in the country, with the majority in the Paktika Province, in the southern region [29]. To eradicate poliovirus from the country, it is imperative that the polio program reaches children who are chronically missed by vaccination campaigns due to their residence in hard-to-reach areas as well as those who are constantly migrating and fail to receive routine or campaign immunization services. In addition, the immunization program needs further strengthening to provide routine immunization against all three poliovirus types to all children, including in high-risk locations. As the immunization program strengthens, parent-held vaccination cards need to be systematically provided by the immunization program [16].

Children remaining inaccessible for vaccination remains a challenge in stopping the transmission of WPV. A significant number of children in the hard-to-reach and security-compromised areas in the country are being missed from vaccination during SIAs. The national polio eradication program of Afghanistan classifies the districts in the country into four categories as per their access status: category one being fully accessible, two—partially accessible, three—accessible with limitations, and four—fully inaccessible [15]. Although the number of inaccessible children varies from campaign to campaign, over the past two years (2105), access deteriorated, particularly in the eastern and northeastern regions [15].

This study has some limitations. The survey population may not be representative of the entire population of Afghanistan or be generalizable to the greater district or province as we conducted a facility-based survey that might have resulted in an overestimation of seroprevalence of antibody against poliovirus because children who are not reached by immunization activities may be less likely to be taken to health care facilities. Furthermore, there is an issue of potential recall bias in caretaker-reported immunization data. Notwithstanding these limitations, our findings are that the polio eradication program of Afghanistan has been successful in achieving overall high seroprevalence of poliovirus neutralizing antibodies in the parts of the country included in this study, which was conducted in 2017.

## 5. Conclusions

Because the findings of this study reflect that there exists a high proportion of seroprevalence of poliovirus neutralizing antibodies among the children of both age cohorts enrolled in this study, which is indicative of the effectiveness of the polio eradication program in Afghanistan toward achieving its underline goal in making the country polio-free. However, there is low PV2 seroprevalence among the younger age group, as found in this study. In addition, this study identified a cluster of seronegative children in the district Poruns of Nuristan province. Furthermore, there is also an accessibility issue in reaching the children in security-compromised areas and districts with always-low routine immunization coverage. Coupled with this, there is high population mobility in the country. These factors increase the risk of cVDPV2 infection among the younger type-2-naive children who live in these inaccessible yet low-immunization-coverage areas and districts and are part of the mobile population. To further enhance immunization coverage in inaccessible areas, there is a need to focus on developing context-specific strategies to mobilize and sensitize the community to the acceptance of vaccination.

## Figures and Tables

**Table 1:** Childhood vaccination schedule Afghanistan.

S. No.	Age	Vaccines
1	Birth (0–11 months)	BCG
2	Birth (as soon as possible within 14 days of life)	OPV0
3	6 Weeks	Pentavalent 1, OPV1
4	10 Weeks	Pentavalent 2, OPV2
5	14 Weeks	Pentavalent 3, OPV3
6	9 Months	Measles, OPV4
7	18 Months	Measles

**Table 2:** Health facilities in targeted provinces.

Health Facility	6–11 Months	36–48 Months	Total
Balkh Regional Hospital, BULKH	81 (7.6%)	64 (6.2%)	145 (6.9%)
Herat Regional Hospital, FARAH	75 (7.1%)	79 (7.7%)	154 (7.4%)
Ghanzi Provincial Hospital, GHAZNI	72 (6.8%)	72 (7.0%)	144 (6.9%)
Herat Regional Hospital, HERAT	67 (6.3%)	72 (7.0%)	139 (6.7%)
Indira Ghandi Hospital and Kochi (Nomad) Hospital, KABUL	76 (7.2%)	79 (7.7%)	155 (7.4%)
Khost Provincial Hospital, KHOST	75 (7.1%)	75 (7.3%)	150 (7.2%)
Nangarhar Regional Hospital, KUNAR	75 (7.1%)	75 (7.3%)	150 (7.2%)
Kunduz Regional Hospital, KUNDUZ	75 (7.1%)	74 (7.2%)	149 (7.1%)
Nangarhar Regional Hospital, LAGHMAN	77 (7.3%)	75 (7.3%)	152 (7.3%)
Nangarhar Regional Hospital, NANGARHAR	71 (6.7%)	79 (7.7%)	150 (7.2%)
Nangarhar Regional Hospital, NORISTAN	74 (7.0%)	78 (7.6%)	152 (7.3%)
Paktika Provincial Hospital, PAKTIKA	85 (8.0%)	65 (6.3%)	150 (7.2%)
Paktya Provincial Hospital, PAKITA	73 (6.9%)	72 (7.0%)	145 (6.9%)
Bost Hospital Lashkargah, HELMAND	83 (7.8%)	70 (6.8%)	153 (7.3%)

**Table 3:** Baseline characteristics of the study population.

Indicators	6–11 Months	36–48 Months	Total
	*n* = 1068	*n* = 1036	*n* = 2104
Interview Status			
Completed	1059 (99.2%)	1029 (99.3%)	2088 (99.2%)
Incomplete	7 (0.7%)	5 (0.5%)	12 (0.6%)
Refused	2 (0.2%)	2 (0.2%)	4 (0.2%)
Blood sample collected	1058 (99.9%)	1028 (99.9%)	2086 (99.9%)
Language			
Pashtu	767 (72.4%)	712 (69.2%)	1479 (70.8%)
Dari	310 (29.3%)	327 (31.8%)	637 (30.5%)
Nuristani	69 (6.5%)	68 (6.6%)	137 (6.6%)
Female	466 (44.0%)	458 (44.5%)	924 (44.3%)
Child Age (months)	9.4 (1.7)	41.8 (4.8)	25.4 (16.6)
Immunization status			
Not immunized	25 (2.4%)	19 (1.8%)	44 (2.1%)
Partially immunized	276 (26.1%)	66 (6.4%)	342 (16.4%)
Fully immunized	758 (71.6%)	944 (91.7%)	1702 (81.5%)
Received IPV	836 (78.9%)	761 (74.0%)	1597 (76.5%)
Received OPV at checkpoint while traveling	576 (54.4%)	596 (57.9%)	1172 (56.1%)
Live in current residence for 12 months of year	1052 (99.3%)	1025 (99.6%)	2077 (99.5%)

## Data Availability

Data are available upon request.
